# Characterization of the small molecule ARC39, a direct and specific inhibitor of acid sphingomyelinase in vitro[S]

**DOI:** 10.1194/jlr.RA120000682

**Published:** 2020-03-10

**Authors:** Eyad Naser, Stephanie Kadow, Fabian Schumacher, Zainelabdeen H. Mohamed, Christian Kappe, Gabriele Hessler, Barbara Pollmeier, Burkhard Kleuser, Christoph Arenz, Katrin Anne Becker, Erich Gulbins, Alexander Carpinteiro

**Affiliations:** * Department of Molecular Biology, University of Duisburg-Essen, 45147 Essen, Germany; † Department of Toxicology, Institute of Nutritional Science, University of Potsdam, 14558 Nuthetal, Germany; § Institute of Chemistry, Humboldt University of Berlin, 12489 Berlin, Germany; ** Medicinal Chemistry Department, Faculty of Pharmacy, Assiut University, Assiut 71526, Egypt; †† Department of Surgery, University of Cincinnati, Cincinnati, OH 45229; §§ Department of Hematology, University Hospital Essen, 45147 Essen, Germany

**Keywords:** sphingolipids, sphingomyelin, ceramides, lipid metabolism, enzymology, lysosome, lysosomal hydrolases, acid ceramidase, bisphosphonates, functional inhibitors of acid sphingomyelinase, 1-aminodecylidene bis-phosphonic acid

## Abstract

Inhibition of acid sphingomyelinase (ASM), a lysosomal enzyme that catalyzes the hydrolysis of sphingomyelin into ceramide and phosphorylcholine, may serve as an investigational tool or a therapeutic intervention to control many diseases. Specific ASM inhibitors are currently not sufficiently characterized. Here, we found that 1-aminodecylidene bis-phosphonic acid (ARC39) specifically and efficiently (>90%) inhibits both lysosomal and secretory ASM in vitro. Results from investigating sphingomyelin phosphodiesterase 1 (*SMPD1/Smpd1*) mRNA and ASM protein levels suggested that ARC39 directly inhibits ASM’s catalytic activity in cultured cells, a mechanism that differs from that of functional inhibitors of ASM. We further provide evidence that ARC39 dose- and time-dependently inhibits lysosomal ASM in intact cells, and we show that ARC39 also reduces platelet- and ASM-promoted adhesion of tumor cells. The observed toxicity of ARC39 is low at concentrations relevant for ASM inhibition in vitro, and it does not strongly alter the lysosomal compartment or induce phospholipidosis in vitro. When applied intraperitoneally in vivo, even subtoxic high doses administered short-term induced sphingomyelin accumulation only locally in the peritoneal lavage without significant accumulation in plasma, liver, spleen, or brain. These findings require further investigation with other possible chemical modifications. In conclusion, our results indicate that ARC39 potently and selectively inhibits ASM in vitro and highlight the need for developing compounds that can reach tissue concentrations sufficient for ASM inhibition in vivo.

Acid sphingomyelinase [human: ASM, sphingomyelin phosphodiesterase 1 (*SMPD1*); mouse: Asm, *Smpd1*; hereafter uniformly ASM] is an enzyme located mainly in the lysosomes where it catalyzes the hydrolysis of sphingomyelin, an abundant sphingolipid in eukaryotic membranes, into ceramide and phosphorylcholine with a pH optimum at ∼5. Ceramide and ASM-generated ceramide are important in cell signaling with versatile roles in many physiological and disease-related processes, particularly when ASM is translocated onto the outer leaflet of the plasma membrane upon a variety of stimuli (1–5). Hereditary mutations of ASM lead to the lysosomal storage disease, Niemann-Pick disease (types A and B), which is characterized by progressive accumulation of sphingomyelin and subsequent organ dysfunction (6, 7). Despite the importance of ASM baseline activity for homeostasis, both genetic deficiency and pharmacological inhibition of ASM have been shown in preclinical studies to ameliorate disease progression in various disease models, with implications in cystic fibrosis (8), hematogenous cancer metastasis (9), atherosclerosis (10), demyelination and myelin repair (11), and autoimmune arthritis (12).

ASM is a phosphodiesterase enzyme that consists of an N-terminal saposin domain and a C-terminal catalytic domain. The saposin domain is responsible, among others, for membrane docking by electrostatic interactions between the positively charged residues on the α2 helix and the anionic lipids in the intra-lysosomal vesicles, like bis(monoacylgycero)phosphate (13). This interaction is important for stabilizing ASM in the proteolytic environment of the lysosome and also greatly affects the conformation and the catalytic activity of the enzyme (13).

Pharmacological inhibition of ASM has, so far, been mostly achieved by applying functional inhibitors of ASM (FIASMAs), which represent a wide spectrum of heterogenous compounds that share certain physiochemical and other properties making them lysosomotropic. Due to their positive charge in the lysosomal acidic pH, FIASMAs are capable of interfering with the electrostatic interaction between the saposin domain of ASM and lysosomal anionic phospholipids, thereby leading to the detachment and deactivation of the enzyme and its proteolytic degradation (14–18). Thus, FIASMAs only inhibit ASM in living cells or in the context of liposomal membranes. Most of these compounds, e.g., the tricyclic antidepressants amitriptyline and desipramine, are clinically approved for the treatment of many conditions, in particular major depressive disorder, and are generally well-characterized. However, FIASMAs, at least in vitro, lack specificity and were shown to inhibit other important lysosomal hydrolases due to the indirect mechanism of action, like acid ceramidase (AC) (19, 20), lysosomal acid lipase (21), and phospholipases A and C, leading to phospholipidosis (22–25). In vitro, they may also upregulate cathepsin L (19) and are capable of permeabilizing the lysosomal membrane and affecting lysosomal stability (26). Many of these effects require much higher doses than necessary for the inhibition of the ASM and are not reached in vivo. In vivo, FIASMAs lead to a reduction of ceramide related to the inhibition of the ASM and have been successfully used to reproduce the phenotype observed in ASM heterozygous mice in models of cystic fibrosis and major depression (4, 5).

In search of direct and high-affinity inhibitors, a bisphosphonate (BP) compound, 1-aminodecylidene bis-phosphonic acid (ARC39; IC_50_ = 20 nM) (**Fig. 1A**), has been developed by Arenz (27) and Roth et al. (28). In this study, we aim to characterize ARC39 in vitro and in vivo in order to provide useful input about its potential application as a direct and specific ASM inhibitor.

**Fig. 1.**
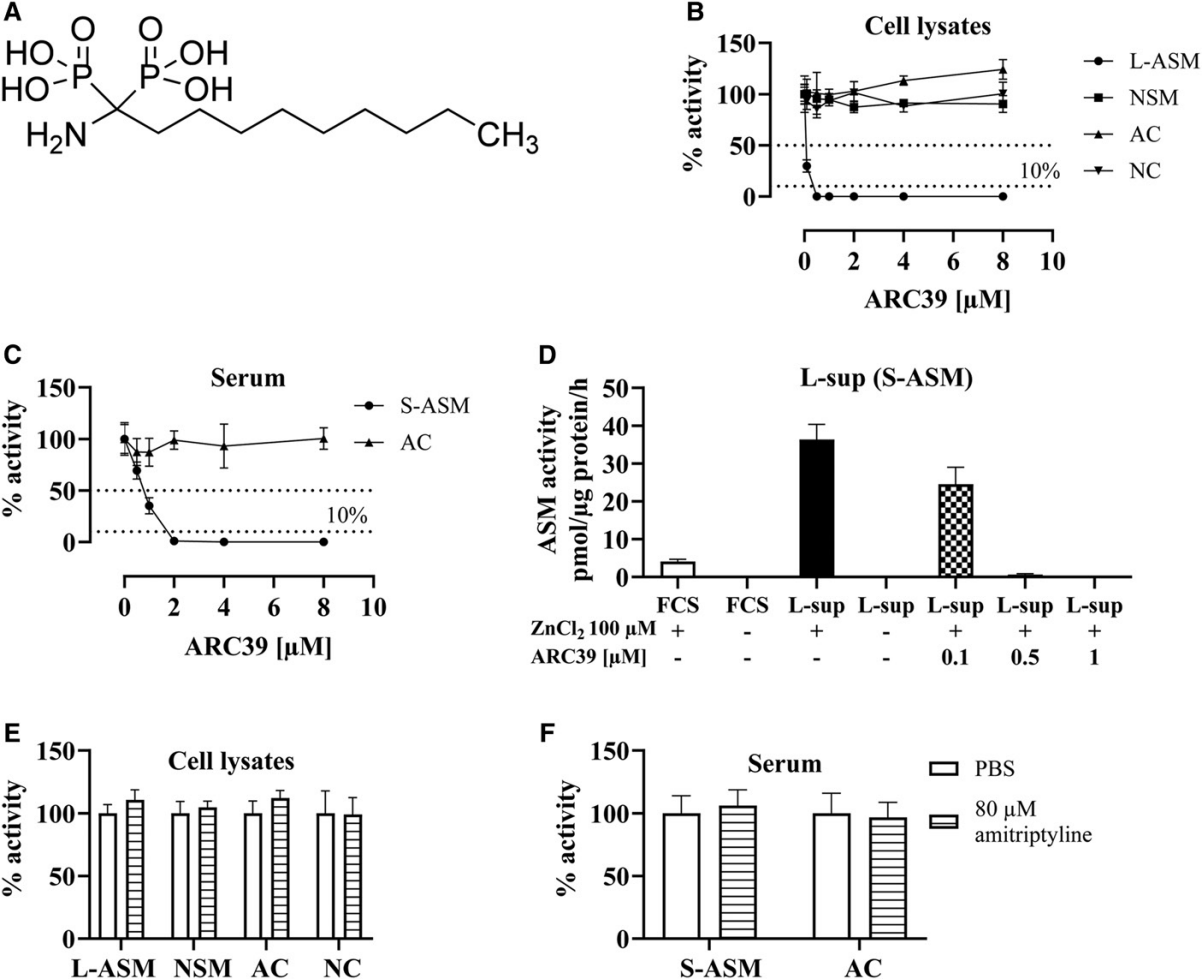
Direct and selective inhibition of L-ASM and S-ASM by ARC39 in micellar assays. A: Chemical structure of ARC39. B: L929 whole-cell lysates were treated with ARC39 as indicated, and the activity of L-ASM (no ZnCl_2_ was added to the assay buffer), NSM, AC, and NC was determined. C: Mouse serum was treated with ARC39 as indicated and the activity of S-ASM (+100 μM ZnCl_2_ in the assay buffer) and AC was determined. D: L929-derived supernatant medium (L-sup) and FCS were treated as indicated with or without addition of ZnCl_2_ to the assay buffer and the activity of S-ASM was determined. E, F: L929 cell lysates or mouse serum, respectively, were treated with PBS control or 80 μM of amitriptyline and the activity of ASM (L-ASM in cell lysates and S-ASM in serum), NSM, AC, and NC was determined. Enzyme activity is normalized to the protein concentration and control values are normalized to their mean except in D where they are expressed as absolute values. Data are represented as mean ± SD, n = 3 experiments.

## MATERIALS AND METHODS

### Cell culture

L929, HepG2, and B16F10 cells (all from ATCC) were cultured in MEM supplemented with 10% FCS, 100 U/ml penicillin, 100 μg/ml streptomycin, 2 mM _L_-glutamine, 1 mM sodium pyruvate, and 100 μM nonessential amino acids (all from Gibco). Cells were maintained at 37°C and 5% CO_2_ in a humidified incubator and were used until passage 20. Cells were grown to subconfluency before experiments.

### Mice

All mice were C57BL/6 Harlan WT female mice and 8–12 weeks old. One gender was used due to the difference in the basal ASM activity between male and female mice, with female mice tending to have higher ASM activity. All mice were bred and housed in the vivarium of the University of Duisburg-Essen, Germany. All mice were pathogen-free according to the 2002 recommendations of the Federation of European Laboratory Animal Science Associations (FELASA). All procedures performed on mice were approved by the Animal Care and Use Committee of the Bezirksregierung Düsseldorf, Düsseldorf, Germany.

### ASM inhibitors

ARC39 was dissolved at 1 mM in PBS and sonicated in a water bath for 2 h with alternating vortexing. The solution was stored in the dark at RT. Amitriptyline hydrochloride (Sigma, A8404) and desipramine hydrochloride (Sigma, D3900) were always prepared as fresh solutions at 1 mM in PBS.

### Conventional ASM, neutral sphingomyelinase, and neutral ceramidase enzyme assays

Cells were seeded in 24-well plates and left to rest overnight before treatment was started. Cells were washed twice with ice-cold PBS. For ASM assay, cells were lysed on ice in ASM lysis buffer [250 mM sodium acetate, 1% NP-40 (IGEPAL, Sigma) (pH 5.0)] for 10 min. For neutral sphingomyelinase (NSM) and neutral ceramidase (NC) assays, cells were collected in a neutral buffer [200 mM HEPES, 0.1% NP-40 (pH 7.0)]. Protein concentration was determined using the Bradford protein reagent (Bio-Rad). BODIPY FL C_12_-sphingomyelin (Invitrogen) and NBD-C_12_-ceramide (Cayman Biochemical) were dissolved at a final concentration of 0.5 μM corresponding to 100 pmol/sample in the corresponding assay buffer: ASM assay buffer {250 mM sodium acetate, 0.1% NP-40, 100 μM ZnCl_2_ [only for secretory ASM (S-ASM)] (pH 5.0)}, NSM assay buffer as previously described (29) [200 mM HEPES, 200 mM MgCl_2_, 0.05% NP-40 (pH 7.0)], NC assay buffer (29) [200 mM HEPES, 100 mM NaCl, 0.03% NP-40 (pH 7.0)]. Substrate solutions and samples were sonicated in a water bath for 10 min. The reaction mixtures consisted of 20 μl of lysate and 180 μl of assay buffer and were incubated for 0.5–3 h at 37°C with 300 rpm. The reaction was terminated by adding chloroform:methanol (2:1, v/v). Phases were separated by centrifugation at 20,000 *g* for 5 min and the organic phase was collected and dried in a SpeedVac (Thermo Fisher). Dried lipids were resuspended in chloroform:methanol (2:1, v/v) and spotted onto a thin-layer chromatography plate (Macherey-Nagel, Germany). Chromatography was conducted with chloroform:methanol (80:20, v/v) in sphingomyelinase assays and with ethyl acetate:acetic acid (100:1, v/v) in NC assays. The plates were scanned using a Typhoon FLA 9500 (GE Healthcare) and spots were quantified with ImageQuant software (GE Healthcare).

### AC assay with RBM14C12

The fluorogenic AC probe, RBM14C12, was obtained from Dr. Antonio Delgado (Faculty of Pharmacy, University of Barcelona). The assay was done as previously described (30): L929 cells were harvested in 100 μl of 0.2 M sucrose on ice provided with protease inhibitor cocktail (Roche) and then were sonicated with a probe for three rounds, 10 W each for 10 s on ice. Cell homogenates were centrifuged at 15,000 *g* for 3 min. The supernatant was collected, and protein quantification was performed. The enzymatic assay was carried out in black 96-well plates. Briefly, each well contained a mixture of 74.5 μl of 25 mM sodium acetate buffer (pH 4.5) (assay buffer), 0.5 μl of a 4 mM RBM14C12 substrate solution in ethanol (substrate final concentration 20 μM; ethanol final concentration 0.5%), and a fixed amount of protein (cell lysates: 20–40 μg; serum: 100 μg) in a volume of 25 μl of a 0.2 M sucrose solution (for cell lysates) or 25 μl of assay buffer for serum. Negative controls consisted of 25 μl of sucrose or 25 μl of assay buffer. After incubation at 37°C for 6 h (serum 1 h) without agitation, the enzymatic reaction was stopped by adding 50 μl of methanol and 100 μl of 2.5 mg/ml NaIO_4_ fresh solution in 100 mM of glycine/NaOH buffer (pH 10.6) in each well. After 2 h incubation in the dark, the released fluorescence was quantified using a FLUOstar Omega microplate reader (BMG Labtech) (λ_Ex_ 355 nm, λ_Em_ 460 nm). The amount of umbelliferone released was calculated using calibration curves with umbelliferone standard (Sigma).

### In situ ASM assay with sphingomyelinase FAM/BODIPY TR FRET probe

The assay was done as previously reported (31). L929 and HepG2 cells were seeded in 96-well plates and left to rest overnight before the experiment was started. Cells were treated with ARC39, as indicated, with PBS as a control. At the end of the treatment, cells were incubated with the sphingomyelinase FAM/BODIPY TR FRET probe (hereafter, FRET probe) for an additional 30 min (L929) or 1 h (HepG2). The final concentration of the FRET probe in the culture medium was 1 μM (from 1 mM stock in DMSO). After incubation, the medium was removed, and cells were washed once with fresh medium followed by brief trypsinization. Cold fresh medium was added (3:1, v/v) to trypsin, and the plate was kept in the dark on ice. The mean fluorescence intensity (MFI) on the green channel (520 nm), which correlated to the cleavage of the substrate, and MFI on the red channel (700 nm), and thereby the uptake of the substrate was detected with an Attune NxT flow cytometer (Thermo Fisher). A minimum of 10,000 events per sample was acquired. Each experiment included unstained controls. To calculate ASM activity, the background fluorescence was first subtracted from all samples, and then the ratio of green:red fluorescence was taken to correct for differences in substrate uptake. Ratios were then normalized to the average ratio of untreated cells and expressed as percent residual ASM activity.

### SDS-PAGE and immunoblotting

Cells were lysed in RIPA buffer [25 mM Tris-HCl (pH 7.3), 0.1% sodium dodecyl sulfate, 0.5% sodium deoxycholate, 1% NP-40, 125 mM NaCl, 10 mM NaF, 10 mM Na_2_P_2_O_7_, 1 mM Na_3_VO_4_, 10 mM EDTA, 10 μg/ml aprotinin, 10 μg/ml leupeptin] supplemented with 1× complete protease inhibitor cocktail (Roche). SDS-PAGE was performed using 10–30 μg total protein. Proteins were transferred to nitrocellulose membranes blocked 1 h with StartingBlock blocking buffer (Thermo Fisher) and incubated with the appropriate primary antibody overnight at 4°C: anti-AC (4741, ProSci, 1:1,000), anti-ASM (AF5348, R&D, 1:50), anti-Ras-related protein 1A (RAP1A) (398755, Santa Cruz, 1:200), anti-β-actin (4778, Santa Cruz, 1:100,000), and subsequently secondary HRP- or AP-conjugated anti-rabbit or anti-mouse antibodies.

### RT-PCR analysis

Total RNA was isolated with RNeasy mini kit (Qiagen) according the manufacturer’s instructions and cDNA synthesis was performed with GoScript reverse transcription kit (Promega) according to the manufacturer’s instructions. RT-PCR was conducted using PowerUp SYBR Green Master Mix (Applied Biosystems) according to the manufacturer’s instructions in a StepOnePlus system (Applied Biosystems) for a total of 40 cycles, each 15 s at 95°C and 1 min at 60°C. Hypoxanthine phosphoribosyltransferase 1 (*Hprt1/HPRT1*) was used as an endogenous control to calculate ΔΔC_t_. Primers used: *Hprt1*_fwd, 5′-ACAGGCCAGACTTTGTTGGAT-3′; *Hprt1*_rev, 5′-ACTTGCGCTCATCTTAGGCT-3′; *Smpd1*_fwd, 5′-TAACCCTGGCTACCGAGTTT-3′; *Smpd1*_rev, 5′-TTGGCCTGGGTCAGATTCAA-3′; *HPRT1*_fwd, 5′-CCCTGGCTGCTCAGTTCTTT-3′; *HPRT1*_rev, 5′-TGGTACACACGGTAACCAGG-3′ (all from Eurofins). *SMPD1* primers were from RT^2^ qPCR Primer Assay for Human *SMPD1* (330001, Qiagen).

### BODIPY sphingomyelin staining

Cells were treated as indicated together with 1 μM of BODIPY FL C_12_-sphingomyelin for 24 h. For confocal microscopy, LysoTracker Red DND-99 (Invitrogen) was added at 25 nM for an additional 30 min; the medium was subsequently changed, and the cells were observed under a Leica TCS SP5 confocal microscope.

### Preparation of mouse plasma and tissues for mass spectrometric analysis

Peripheral blood was collected from the retro orbital plexus into EDTA tubes on ice. The tubes were centrifuged at 1,700 *g*, 4°C for 10 min. Plasma was collected and centrifuged again at 3,000 *g*, 4°C for 5 min and stored at −80°C until use. Peritoneal lavage was collected by washing the abdominal cavity with a total of 10 ml of cold PBS followed by centrifugation at 300 *g*, 4°C for 5 min. The pellet was resuspended in PBS and an aliquot was taken to determine protein concentration; then the lavage was pelleted again followed by adding 500 μl methanol and stored at −80°C until use. The indicated organs were harvested and snap-frozen in liquid nitrogen followed by preparing organ powder in liquid nitrogen with a pestle and mortar. The powder was stored at −80°C until use.

### Sphingolipid quantification by HPLC-MS/MS

Cell, tissue, and plasma samples were subjected to lipid extraction using 1.5 ml methanol/chloroform (2:1, v:v) as described (32). The extraction solvent contained C17-dihydrosphingosine (C17 dhSph), d_7_-sphingosine (Sph), d_7_-Sph-1-phosphate (S1P), C17-ceramide (Cer17), and C16-d_31_-sphingomyelin (d_31_-SM16) (all Avanti Polar Lipids, Alabaster, AL) as internal standards. Chromatographic separations were achieved on a 1260 Infinity HPLC (Agilent Technologies, Waldbronn, Germany) equipped with a Poroshell 120 EC-C8 column (3.0 × 150 mm, 2.7 μm; Agilent Technologies). A mobile phase system consisting of water (solvent A) and acetonitrile/methanol (1:1, v:v; solvent B), both acidified with 0.1% formic acid, was used for gradient elution at an initial composition of 40:60 (A:B, v:v) and a flow rate of 0.5 ml/min. MS/MS analyses were carried out using a 6490 triple-quadrupole mass spectrometer (Agilent Technologies) operating in the positive electrospray ionization mode (ESI+). The following ion source parameters were set: sheath gas temperature, 375°C; sheath gas flow, 12 l/min of nitrogen; nebulizer pressure, 30 psi; drying gas temperature, 200°C; drying gas flow, 15 l/min of nitrogen; capillary voltage, 4,000 V; nozzle voltage, 1,500 V; iFunnel high pressure RF voltage, 150 V, and iFunnel low pressure RF voltage, 60 V. The following mass transitions were recorded [collision energies (CEs) in parentheses]: long-chain bases: *m/z* 288.5 → 270.5 for C17 dhSph (12 eV), *m/z* 300.3 → 282.3 for Sph (8 eV), *m/z* 302.3 → 284.3 for dhSph (6 eV), *m/z* 307.3 → 289.3 for d_7_-Sph (8 eV), *m/z* 380.3 → 264.3 for S1P (16 eV), and *m/z* 387.3 → 271.3 for d_7_-S1P (16 eV); ceramides (CE = 25 eV for all transitions): *m/z* 520.5 → 264.3 for Cer16, *m/z* 534.5 → 264.3 for Cer17, *m/z* 548.5 → 264.3 for Cer18, *m/z* 576.6 → 264.3 for Cer20, *m/z* 604.6 → 264.3 for Cer22, *m/z* 630.6 → 264.3 for Cer24:1, and *m/z* 632.6 → 264.3 for Cer24; sphingomyelins (CE = 25 eV for all transitions): *m/z* 703.6 → 184.1 for SM16, *m/z* 731.6 → 184.1 for SM18, *m/z* 734.8 → 184.1 for d_31_-SM16, *m/z* 759.6 → 184.1 for SM20, *m/z* 787.7 → 184.1 for SM22, *m/z* 813.7 → 184.1 for SM24:1, and *m/z* 815.7 → 184.1 for SM24. The dwell time for all mass transitions recorded was 75 ms. Quantification was performed with MassHunter Software (Agilent Technologies). Depending on the sample type analyzed sphingolipid contents were normalized to cell count or protein (as determined by Bradford assay).

### Sph kinase 1 and Sph kinase 2 assays

NBD-C_18_-shingosine (Avanti Polar Lipids) was prepared and dissolved in 5% Triton X-100, and Sph kinase (SphK1) and SphK2 assays were performed in real-time as previously described (33) with slight changes. Briefly, 96-well polypropylene plates were used. Fluorescence emission was measured with a FLUOstar Omega (BMG Labtech). Excitation wavelength was 544 nm, and emission wavelength was 590 nm. Assays were initiated with 20× ATP [20 mM ATP, 200 mM MgCl_2_, 900 mM Tris-HCl (pH 7.4)] followed by shaking and mixing for 15 s. Assays were prepared as master mixes immediately before use in either SphK1 or SphK2 reaction buffer containing 30 μM of NBD-Sph, 150 nM of rhSphK1 (R&D Systems, 5536-SK-010), or 6.9 nM of rhSphK2 (R&D Systems, 5298-SK-010). ARC39 was added as indicated. SphK1 reaction buffer contained 30 mM of Tris-HCl (pH 7.4), 0.05% Triton X-100, 150 mM of NaCl, 10% glycerol, 1 mM of Na_3_VO_4_, 10 mM of NaF, and 10 mM of β-glycero-phosphate. SphK2 reaction buffer contained 30 mM of Tris-HCl (pH 7.4), 0.05% Triton X-100, 200 mM of KCl, and 10% glycerol.

### Mouse platelet isolation

As previously described (9), blood was collected by tail vein puncture and anti-coagulated with 0.38% sodium citrate, and 9 ml of PBS (pH 7.2) supplemented with 3.5% BSA, fatty acid free (Sigma-Aldrich) were added, and this mixture was incubated for 15 min at 37°C. Samples were centrifuged at 120 *g* for 20 min without stopping at RT, and the platelet-containing supernatant was collected. Platelets were pelleted by centrifugation with 1,340 *g* for 10 min and were resuspended in tyrode’s buffer (134 mM of NaCl, 0.34 mM pf Na_2_HPO_4_, 2.9 mM pf KCl, 12 mM of NaHCO_3_, 20 mM of HEPES, 5 mM of glucose). After preparation, platelets were used immediately for the respective experiments in the indicated concentrations.

### Preparation of tumor cells

Cells were brought into suspension by treatment with cell dissociation solution [enzyme-free PBS-based (Gibco)], washed extensively, and resuspended in HEPES/saline [132 mM of NaCl, 20 mM of HEPES (pH 7.4), 5 mM of KCl, 1 mM of CaCl_2_, 0.7 mM of MgCl_2_, and 0.8 mM of MgSO_4_] in the indicated concentration.

### Adhesion assays

As previously described (9), B16F10 melanoma cells and platelets were prepared simultaneously as described above. 4 × 10^4^ B16F10 tumor cells were then incubated with 2 × 10^7^ WT or Asm-deficient platelets or 0.125 μg rhASM (R&D Systems, 5348-PD-010) for 5 min in a total volume of 100 μl at 37°C. After the addition of 300 μl of complete culture medium, tumor cells were transferred to fibronectin-coated glass cover slips (fibronectin from Roche, 11051407001) in 24-well plates and incubated for 3 min at 37°C. Unbound tumor cells were washed away four times with HEPES/saline buffer, and cells were fixed with 2% buffered PFA for 15 min. After being washed three times with PBS, cells were stained with 1 μg/ml DAPI (Sigma) in PBS for 15 min at RT and washed with PBS. Adherent cells per coverslip (diameter 12 mm) were counted with an EVOS FL imaging system (Thermo Fisher).

### Cell death and viability

To assess cell death, cells were harvested, washed once with Annexin V Binding Buffer (BD Bioscience) after the indicated treatment and incubated for 15 min at RT with APC Annexin V (Biolegend) 1:20/propidium iodide (Sigma) 1 μg/ml in annexin V binding buffer. Data acquisition and analysis were performed with an Attune NxT flow cytometer (Thermo Fisher). A minimum of 10,000 events per sample was acquired. Cell viability was determined with XTT Cell Viability Assay Kit (Biotium) according to the manufacturer’s instructions.

### Lysosomal staining

After the indicated treatments, either LysoTracker Red DND-99 final concentration 25 nM or LysoSensor Blue DND-167 final concentration 10 μM (both Invitrogen) was added and incubated at 37°C for 30 min. For confocal microscopy, the medium was replaced with fresh medium and cells were observed under a Leica TCS SP5 confocal microscope. For flow cytometric analysis, cells were trypsinized and MFI was analyzed with an Attune NxT (Thermo Fisher). A minimum of 10,000 events per sample was acquired.

### Detection of phospholipidosis

HCS LipidTOX Green Phospholipidosis Detection Reagent (Invitrogen) was diluted 1:1,000 with the complete culture medium, and then the medium was added to the cells. This reagent characterizes potentially toxic side effects of compounds on lipid metabolism in mammalian cells, and the fluorescently labeled phospholipids accumulate if the test compound induces phospholipidosis. Cells were then treated as indicated and incubated for 24 h. For confocal microscopy, the medium was replaced with fresh medium and the cells were observed under a Leica TCS SP5 confocal microscope. For flow cytometric analysis, cells were trypsinized and MFI was analyzed with an Attune NxT (Thermo Fisher). A minimum of 10,000 events per sample was acquired.

### Mouse blood chemistry analysis

Peripheral blood was collected from the lateral tail vein and left 1.5–2 h at RT to coagulate followed by centrifugation at 2,500 *g* at RT for 20 min. Serum was collected and centrifuged again at 2,500 *g* for 5 min.

Serum was analyzed by using SpotChem EZ chemistry analyzer with the corresponding parameter strips (Scil, Viernheim, Germany). Parameters determined were: blood urea nitrogen (single strips), aspartate transaminase (liver-1 strips), alanine transaminase (liver-1 strips), lactate dehydrogenase (liver-1 strips), creatine phosphokinase (single strips), and amylase (single strips).

### Statistical analyses

Statistical analysis was performed using GraphPad Prism (GraphPad Software). Data are expressed as arithmetic means ± standard deviation. We used two-tailed unpaired Student’s *t*-test for two-group comparisons, and one-way or two-way ANOVA with Tukey’s or Bonferroni post hoc test for multiple comparisons. Values of *P* < 0.05 were considered significant. All data were obtained from independent measurements.

## RESULTS

### ARC39 inhibits both lysosomal ASM and S-ASM directly and specifically in micellar assays

ARC39 has been reported to have a direct mode of action on the recombinant ASM by binding at the active site and thereby blocking the substrate binding and also excluding the nucleophilic water molecule (13). ASM is known to have a lysosomal form and a secretory form that arise from the same polypeptide precursor due to differential glycosylation. Lysosomal ASM (L-ASM) activity is detectable in cell and tissue lysates without the need to add zinc to the assay buffer, while the activity of S-ASM is detectable in sera, body fluids, and cellular supernatant media, and usually requires addition of zinc to the assay buffer to activate the enzyme (also recombinant ASM). In a first approach, we wanted to confirm the direct interaction of the inhibitor with ASM. To this end, we treated L929 whole-cell lysates (L-ASM), L929-derived supernatant medium (S-ASM), and mouse serum (S-ASM) directly with ARC39. No zinc was added to the assay buffer of L-ASM, while the activity of S-ASM from L929 medium, FCS, and mouse serum was not detectable without further addition of zinc (Fig. 1D) (mouse serum without zinc not shown). We found an efficient dose-dependent inhibition of L-ASM activity (Fig. 1B) and S-ASM activity in mouse serum and L929 medium (Fig. 1C, D), respectively. In contrast, amitriptyline, a tricyclic antidepressant and a FIASMA, as already known due to its indirect mode of action, does not inhibit ASM in this setting neither in cell lysates nor in serum even when applied at up to 80 μM (Fig. 1E, F), which is a high dose that is far above the dose used to treat cells. To confirm the enzyme specificity of ARC39, we additionally investigated its direct effect on AC, NC, and NSM in this same setting. No reduction in the activities of these enzymes was detected (Fig. 1B, C).

### ARC39 inhibits L-ASM directly and specifically in cultured cells

**Fig. 2.**
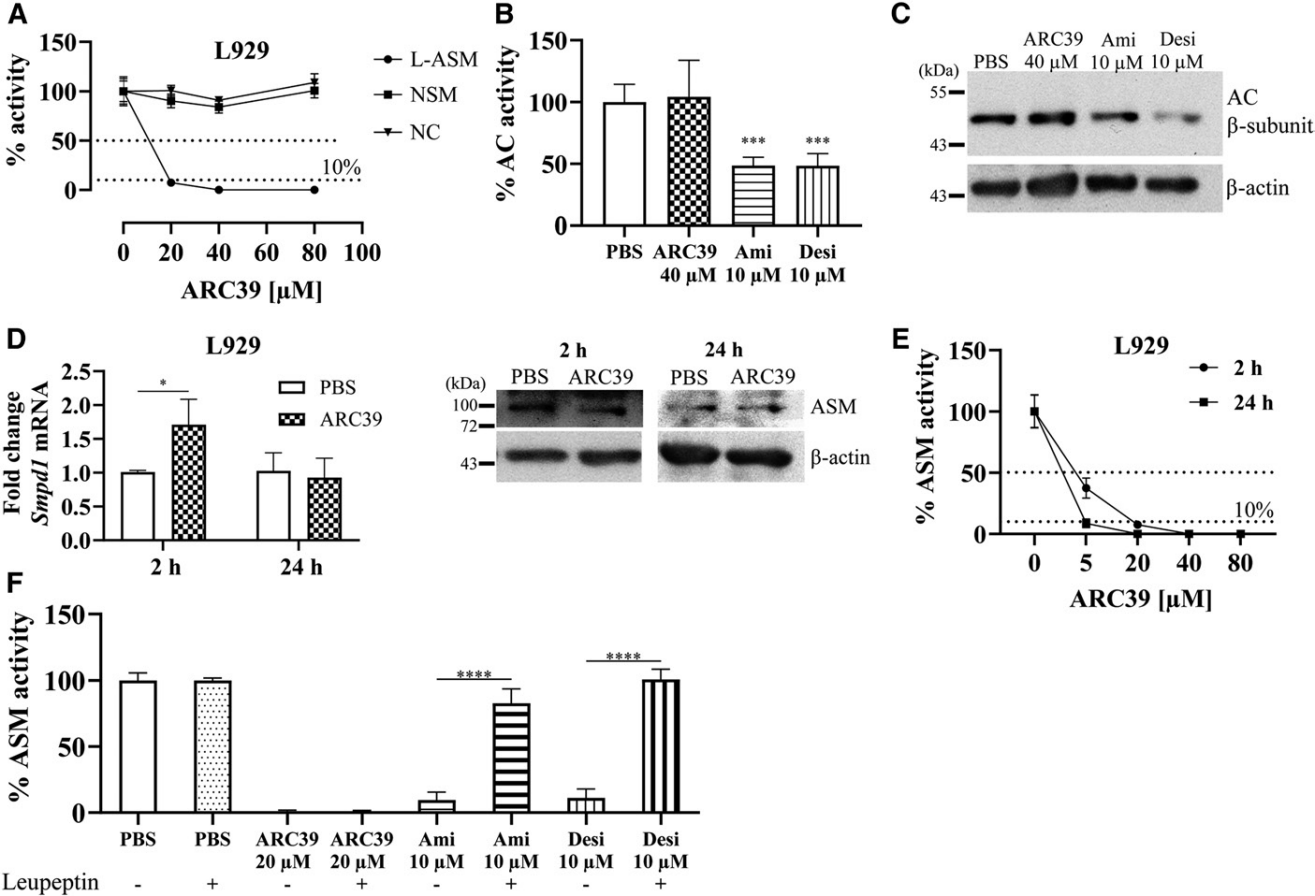
Direct and specific inhibition of ASM by ARC39 under cell culture conditions. A: L929 cells were treated for 2 h with ARC39 as indicated, and the activity of ASM, NSM, and NC was determined. L929 cells were treated for 2 h with ARC39, amitriptyline (Ami) or desipramine (Desi) as indicated, and AC activity was subsequently determined in B, and AC protein was analyzed by Western blotting in C. D: L929 cells were treated with 20 μM of ARC39 as indicated: fold change of *Smpd1* mRNA relative to *Hprt1* (left), ASM protein level (representative Western blot) (right). E: ASM activity in L929 cells after treatment with ARC39 as indicated. *P* < 0.01 for treatment time as a source of variation in ASM activity. F: L929 cells were preincubated for 24 h with 25 μM of leupeptin or vehicle then treated for 4 h, as indicated, and ASM activity was subsequently determined. Enzyme activity is normalized to the protein concentration, and control values are normalized to their mean. ASM activity shown in this figure is determined with the conventional assay without addition of zinc and is attributed to L-ASM. Data are represented as mean ± SD, n = 3–5 experiments. One-way ANOVA was used in B and F and two-way ANOVA in D and E; both were followed by Bonferroni correction (except for ASM activity). *P < 0.05, ***P < 0.001, ****P < 0.0001.

Next, ARC39 was tested under cell culture conditions. L929 murine fibroblasts, with an abundant ASM activity compared with e.g., HepG2 (personal observations, data not shown), were treated with increasing doses of ARC39 and the activity of ASM, NSM, AC, and NC was determined. ARC39 inhibited ASM efficiently in a dose-dependent manner while having no inhibitory effect on the other enzymes (**Fig. 2A**, B). The commonly used ASM inhibitors desipramine and amitriptyline efficiently inhibited ASM under these conditions as well (Fig. 2F) but also, and in contrast to ARC39, reduced AC activity (Fig. 2B) subsequent to the degradation of AC protein (Fig. 2C).

To investigate or exclude other potential mechanisms of ASM inhibition in cells, such as downregulating mRNA or ASM protein, we followed *SMPD1*/*Smpd1* mRNA and ASM protein expression levels 2 and 24 h after treatment with ARC39 in L929 and HepG2 cells (Fig. 2D, supplemental Fig. S1), respectively. *SMPD1*/*Smpd1* mRNA was not downregulated. In contrast, in L929 cells, we noticed a transient upregulation 2 h after treatment with ARC39 (Fig. 2D). The ASM protein level was not significantly changed, although the enzyme activity was not detectable (Fig. 2D, E; supplemental Fig. S1). Desipramine was reported to induce the proteolytic degradation of ASM, and this effect was prevented by pretreatment with the serine/cysteine protease inhibitor leupeptin (18). Preincubating cells with leupeptin abrogated ASM inhibition induced by amitriptyline and desipramine but not by ARC39 (Fig. 2F).

Taken together, these data suggest that direct inhibition of ASM catalytic activity is also the mechanism by which ARC39 inhibits ASM in cultured cells. Of note, ARC39 has the following values: log *P* = 3.35, p*K*_a_ (non-ionized) = 2.179 (calculated with ChemDraw 8.0, CambridgeSoft Corporation). These values differ clearly from those reported for FIASMAs (log *P* = 5.35 ± 1.13, p*K*_a_ = 9.04 ± 1.18) (14).

### ARC39 inhibits L-ASM dose and time dependently in intact living cells

While the conventional ASM assay used here is quite useful and reliable when investigating changes in activity subsequent to alterations in ASM protein itself (including proteolytic degradation upon treatment with FIASMAs) both in cell and tissue lysates (29), it may not be as reliable when investigating inhibitors with a direct mode of action because of the many processing and dilution steps after initial treatment, which may affect the interaction between the enzyme and the inhibitor. Thus, the values obtained in such an assay may not reflect the actual inhibition. Very recently, a new FRET probe-based in situ ASM assay was developed and validated (31). The FRET probe is a dual-labeled sphingomyelin in which FAM acts as FRET donor and BODIPY as FRET acceptor. Cleavage of the probe results in a slight decrease in BODIPY fluorescence and a marked increase in FAM fluorescence. This ratiometric assay utilizes flow cytometry to obtain a dose-response in intact cells: The FRET probe emits green only upon cleavage, while the red fluorescence can be used to correct for differences in the uptake of the probe by cells. Notably, there is an inherent difference between this assay and the conventional assay: the in situ assay with the FRET probe tends to give higher residual activity values, and there is inevitably 5–10% residual activity (31).

**Fig. 3.**
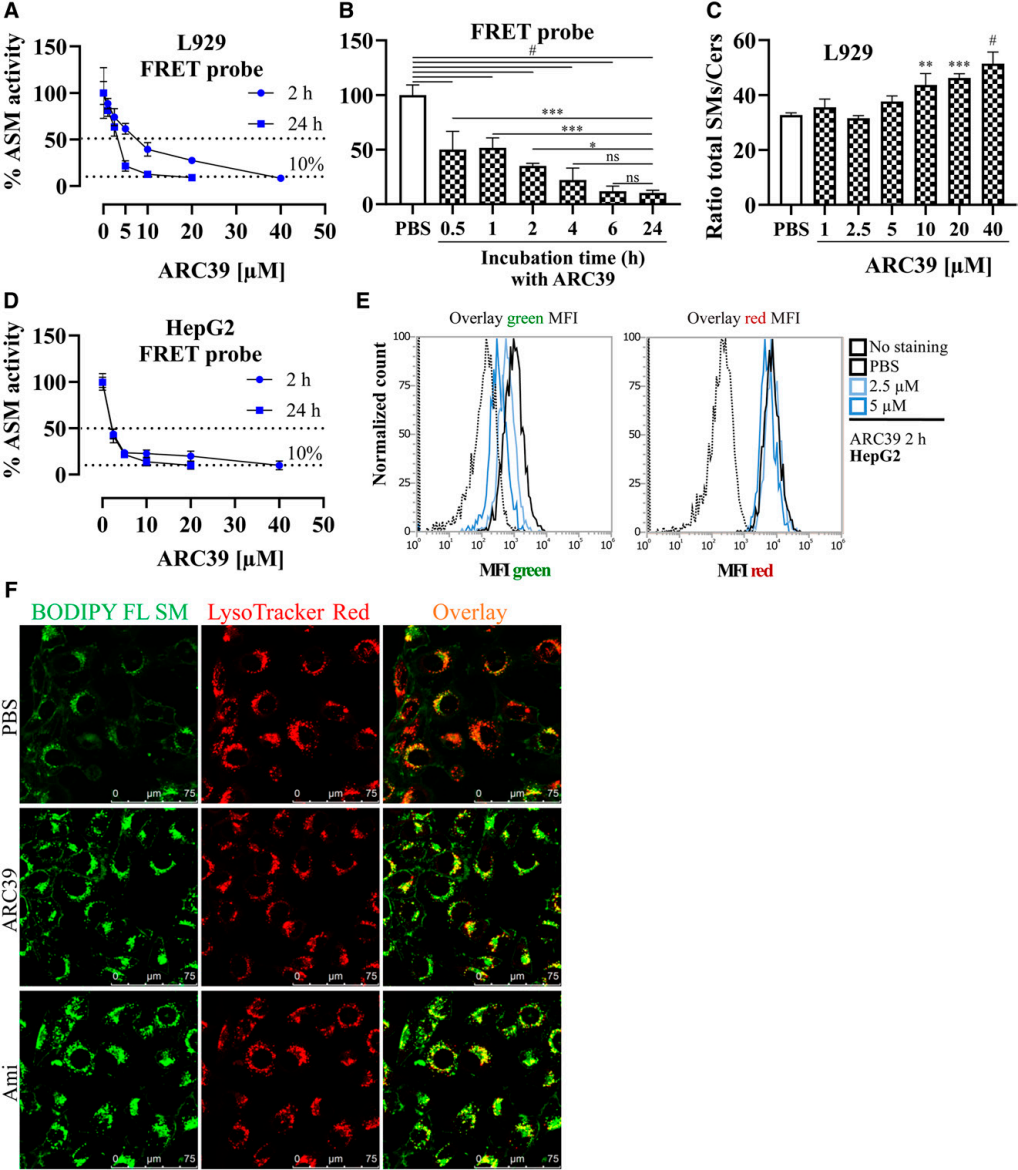
Inhibition of L-ASM in living cells by ARC39. A, B, D: After the indicated treatments for the indicated durations, cells were incubated with the FRET probe at a final concentration of 1 μM for 30 min (L929) or 1 h (HepG2) and were then washed, briefly trypsinized, and analyzed by flow cytometry. A: Comparison of the dose response in L929 cells treated with ARC39 for 2 h or 24 h. *P* < 0.0001 for time as a source of variation in ASM activity. B: Effect of the treatment duration on ASM inhibition by ARC39. L929 cells were treated with 20 μM of ARC39 for the indicated times, and then ASM activity was determined with the FRET probe. C: Mass spectrometric analysis of the ratio of total sphingomyelins/total ceramides (SMs/Cers) in whole-cell lysates from L929 cells that had been treated for 24 h with ARC39 as indicated. D: Comparison of the dose-response in HepG2 cells treated with ARC39 for 2 h or 24 h. *P* < 0.05 for time as a source of variation in ASM activity. E: Representative histogram overlay for the MFI of the FRET probe in both green (520 nm) and red (700 nm) channels from HepG2 cells treated as indicated for 2 h. F: L929 cells were incubated for 24 h with 1 μM of BODIPY FL sphingomyelin (SM) together with PBS as control, 10 μM of ARC39 or 10 μM of amitriptyline (Ami) as a positive control. Then, the cells were incubated for 30 min at 37°C with 25 nM of LysoTracker Red DND-99. Fresh medium was added, and then the cells were analyzed by confocal microscopy (magnification 100×). Control values are normalized to their mean. Data are represented as mean ± SD, n = 3–4 experiments. One-way ANOVA was used in B and C followed by Bonferroni correction, and two-way ANOVA was used in A and D. *P < 0.05, **P < 0.01, ***P < 0.001, #*P* < 0.0001.

Using this assay, ARC39 led to an efficient dose- and time-dependent inhibition of ASM activity in intact L929 cells (**Fig. 3A**, B). The inhibitory effect started within 30 min and reached its maximum at ∼6 h after treatment in L929 cells (Fig. 3B). Moreover, ARC39 led to a dose-dependent increase in the ratio of total sphingomyelins/ceramides in L929 whole-cell lysates after 24 h beginning from 5 μM (significant from 10 μM) as determined by mass spectrometric analysis (Fig. 3C), further corroborating the results obtained with the FRET probe. A dose-response was also obtained in HepG2 cells using the FRET probe (Fig. 3D, E).

Next, we further validated the lysosomal accumulation of sphingomyelin by using exogenous fluorescently labeled sphingomyelin. L929 cells were incubated with BODIPY FL C_12_-sphingomyelin together with 10 μM of ARC39 or 10 μM of amitriptyline as a positive control. In cells treated with either ARC39 or amitriptyline, we detected intracellular accumulation of the exogenous sphingomyelin within aggregates after 24 h, which colocalized with LysoTracker Red (Fig. 3F), indicating that treatment with ARC39 leads to accumulation of sphingomyelin in the lysosomal compartment. Similar lysosomal accumulation of exogenous sphingomyelin was observed in HepG2 cells (supplemental Fig. S2).

### Changes in sphingolipids upon treatment with ARC39

**Fig. 4.**
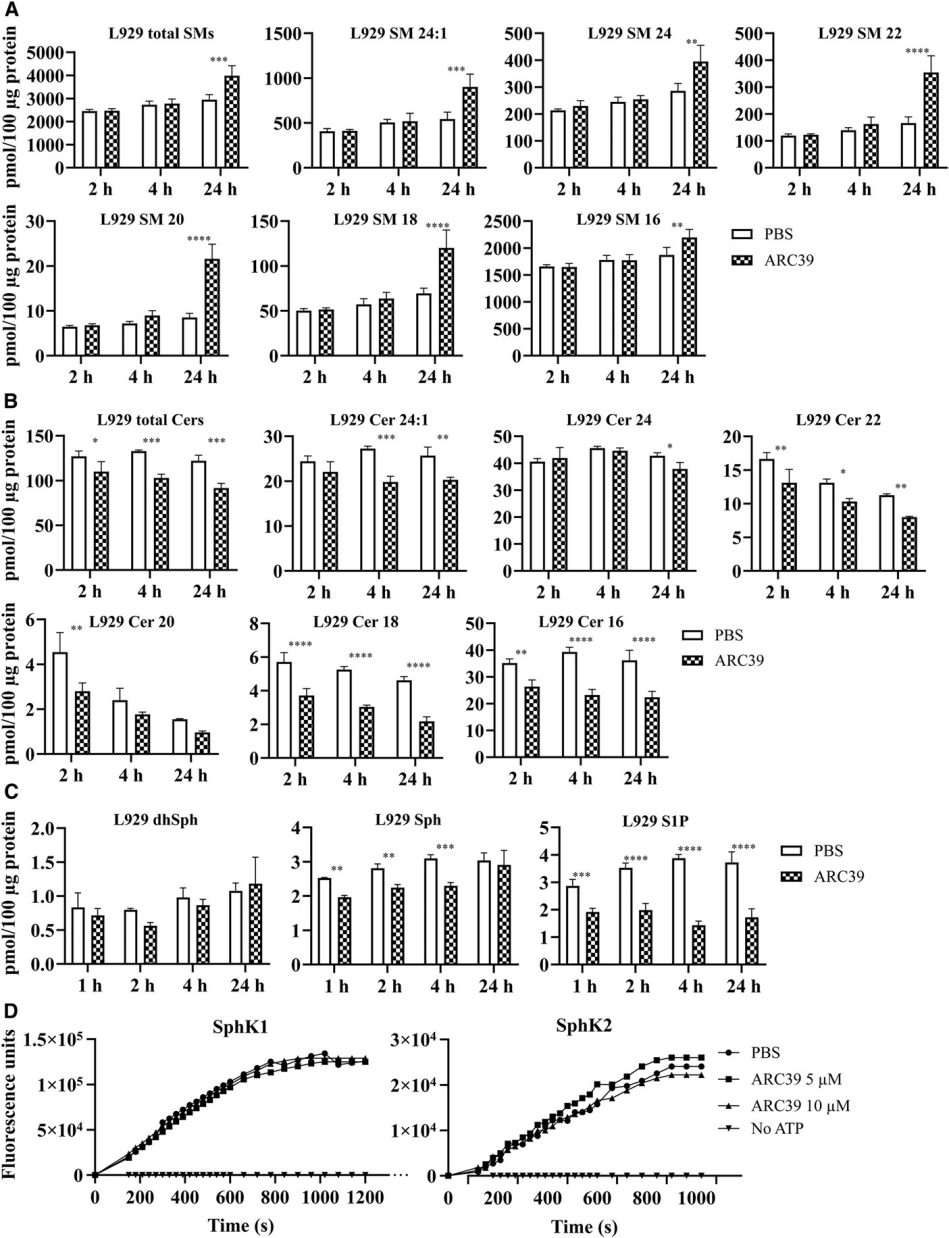
Sphingolipidomic analysis after treatment with ARC39 in L929 cells. Cells were treated with 20 μM of ARC39 for the indicated durations and the endogenous levels of the following sphingolipids were determined by MS in whole-cell lysates: sphingomyelins (SMs) (A); ceramides (Cers) (B). Numbers indicate the chain length and saturation of the fatty acyl chain. C: dhSph, Sph, and S1P, respectively. D: Time-resolved fluorescence emission at 590 nm. Activity of rhSphK1 (left) and rhSphK2 (right) was determined in real-time after pretreatment with ARC39 as indicated. The reaction mixture contained 30 μM of NBD-C_18_-Sph, 150 nM of SphK1, or 6.9 nM of SphK2 with or without 1 mM of ATP. Data are represented as mean ± SD, n = 3 experiments. Two-way ANOVA was used followed by Bonferroni correction. *P < 0.05, **P < 0.01, ***P < 0.001, ****P < 0.0001.

Next, mass spectrometric analysis was undertaken to determine potential changes in endogenous sphingolipids in whole-cell lysates from cells that had been treated with 20 μM of ARC39. In L929 cells, an increase in total sphingomyelins and in all tested sphingomyelin species (C_16_–C_24_) was observed after 24 h (**Fig. 4A**). A faster reduction in total ceramides and in individual C_16_–C_24_ ceramide species was detected within 2 h after treatment (Fig. 4B). Overall, there were no significant changes in dhSph (Fig. 4C) or dihydroceramides (not shown). Sph, however, was reduced downstream of ceramide reduction (Fig. 4C). Interestingly, the intracellular levels of S1P were, albeit in the relatively low picomole range, significantly and time-dependently reduced (Fig. 4C). Mass spectrometric analysis in HepG2 cells yielded similar results (supplemental Fig. S3).

These changes in intracellular S1P prompted us to investigate whether ARC39 could potentially inhibit SphK1 or SphK2. ARC39 was directly incubated with rhSphK1 or rhSphK2 and enzyme activities were subsequently determined. Up to 10 μM ARC39 did not exhibit an inhibitory effect on SphK1 or on SphK2 (Fig. 4D; 0.5–10 μM were tested; for convenience, only 5 and 10 μM are shown). Furthermore, as much as 80 μM of ARC39 had no effect on SphK1 or SphK2 activity when the reaction buffer contained 10 μM of NBD-Sph (not shown).

### ARC39 inhibits platelet- and ASM-promoted adhesion of tumor cells

ARC39 has been used in numerous studies to yield functional results related to the inhibition of ASM activity (28, 34–38).

Our group previously showed that adhesion and metastasis of murine B16F10 melanoma is highly promoted by a secreted zinc-dependent form of ASM that is rapidly released from WT platelets upon interaction with tumor cells due to clustering and activation of α5β1 integrins on the surface of tumor cells in ceramide-enriched platforms (9). Tumor cells are not the source of ASM in this case, and their promoted adhesion in vitro and metastasis in vivo is abrogated upon interaction with Asm^−/−^ platelets (9).

**Fig. 5.**
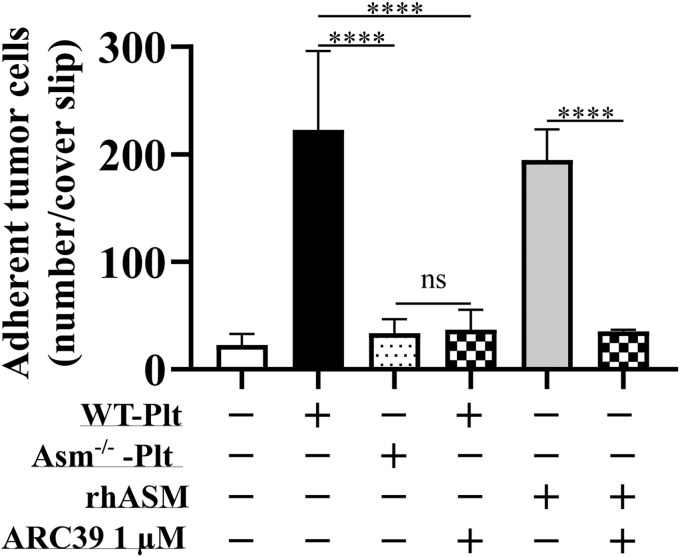
Functional validation of ASM inhibition by ARC39: ARC39 inhibits platelet- and ASM-promoted adhesion of tumor cells. B16F10 melanoma cells (4 × 10^4^) were incubated for 5 min at 37°C in 100 μl total volume with 2 × 10^7^ WT Asm-deficient (Asm^−/−^) platelets (Plts) or rhASM (0.125 μg in 50 μl) in the presence or absence of 1 μM of ARC39, which was added directly upon coincubation without previous pretreatment. After addition of 300 μl of B16F10 MEM complete culture medium (also containing 1 μM of ARC39 where indicated), tumor cells were incubated for 3 min on fibronectin-coated coverslips, washed, fixed, and stained with DAPI. Adherent tumor cells were then counted. Data are represented as mean ± SD, n = 3–4 experiments. One-way ANOVA was used followed by Tukey’s correction. ****P < 0.0001.

To obtain a functional validation in the current study, we incubated B16F10 melanoma cells with WT platelets with or without 1 μM of ARC39, platelets from Asm^−/−^ mice, and rhASM with or without 1 μM of ARC39 and determined the adhesion of melanoma cells to fibronectin-coated cover slips. Both WT platelets and rhASM rapidly promoted the adhesion of melanoma cells. Asm^−/−^ platelets did not promote B16F10 cell adhesion, which was phenocopied by adding 1 μM of ARC39 just before incubating WT platelets with melanoma cells. The same was observed with rhASM (**Fig. 5**).

### Toxicity in vitro

**Fig. 6.**
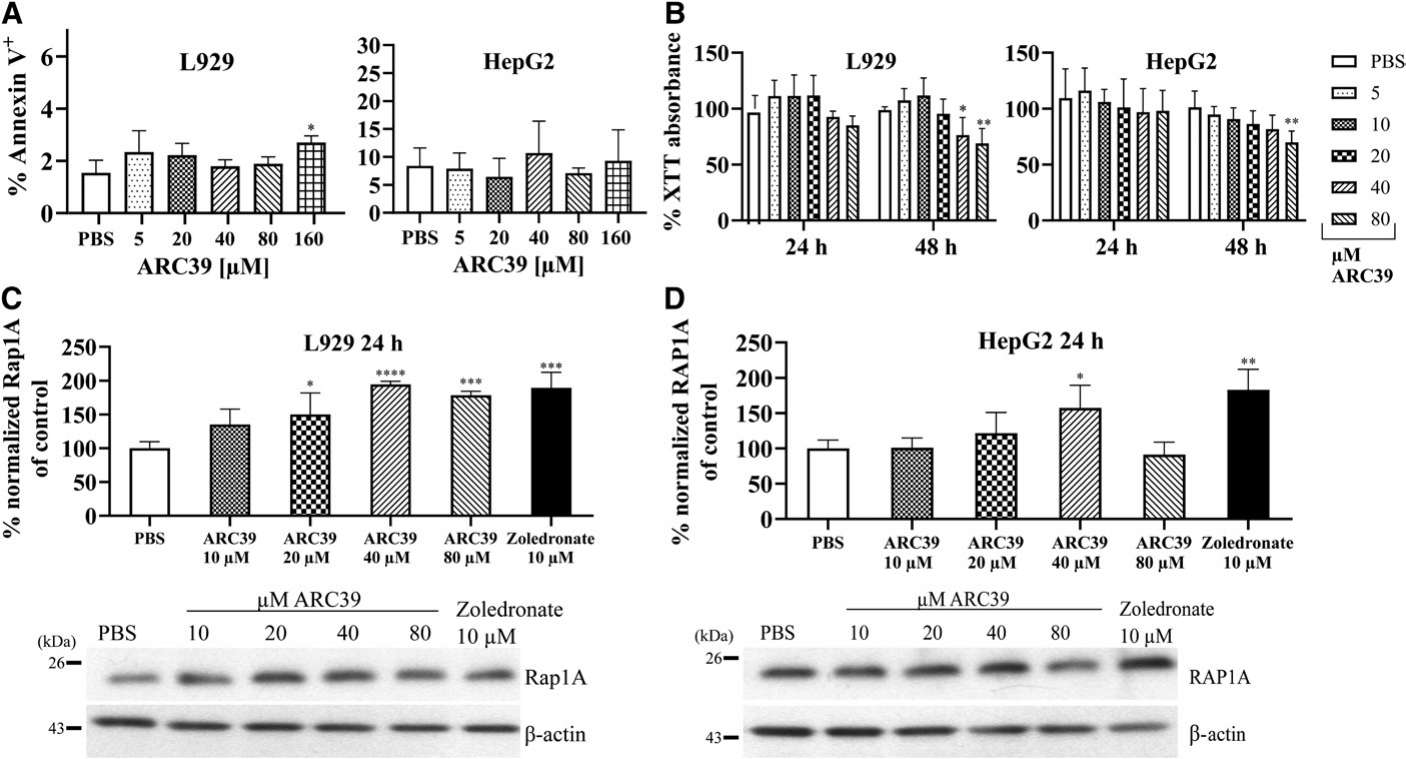
Toxicity of ARC39 in vitro. (A, B) L929 and HepG2 cells were treated with ARC39 as indicated. A: After 24 h treatment, cells were stained with annexin V/PI and analyzed by flow cytometry. B: After 24 and 48 h treatment, cell viability was determined with XTT reduction assay. C, D: Unprenylated Rap1A and RAP1A protein, respectively, normalized to β-actin in L929 and HepG2 cells, respectively, after 24 h treatment with ARC39 with representative Western blots. Zoledronate served as a positive control. Control values are normalized to their mean. Data are represented as mean ± SD, n = 3–4 experiments. One-way ANOVA was used in A, C, and D and two-way ANOVA in B, both followed by Bonferroni correction. *P < 0.05, **P < 0.01, ***P < 0.001, ****P < 0.0001.

In the cells tested (also Jurkat and primary bone marrow-derived macrophages, not shown), no induction of cell death was detected 24 h after incubation with ARC39 even with doses as high as 160 μM, except for L929 that showed a slight increase of the annexin V^+^ population (**Fig. 6A**).

The overall cell viability was not significantly reduced after 24 h with higher doses (Fig. 6B). Toxicity after 48 h appeared in L929 and HepG2 with higher doses (≥40 μM), which are above the range required for ASM inhibition (Fig. 6B).

One known mechanism of toxicity by nitrogen-containing BPs (N-BPs) is the inhibition of farnesyl pyrophosphate (FPP) synthase in the mevalonate pathway (39–42). Thus, we investigated whether ARC39 could potentially inhibit FPP synthase by detecting unprenylated Rap1A/RAP1A protein in L929 and HepG2 cells, respectively, after treatment with increasing doses of ARC39. After 24 h, we detected higher levels of unprenylated Rap1A in L929 compared with vehicle-treated cells, particularly with doses that led to toxicity at 48 h (Fig. 6C). Accumulation of unprenylated RAP1A was also present in HepG2 cells albeit to a lesser extent (Fig. 6D). This suggests that inhibition of FPP synthase could be, at least partially, a potential cause for the toxicity seen at later time points with ARC39.

### Effect of ARC39 on the lysosomal compartment

To this end, L929 cells were treated for 2 h, indicative of a direct effect of the compound on the lysosomes, or for 24 h, indicative of an indirect effect due to accumulation of sphingomyelin and potential adaptive lysosomal responses.

**Fig. 7.**
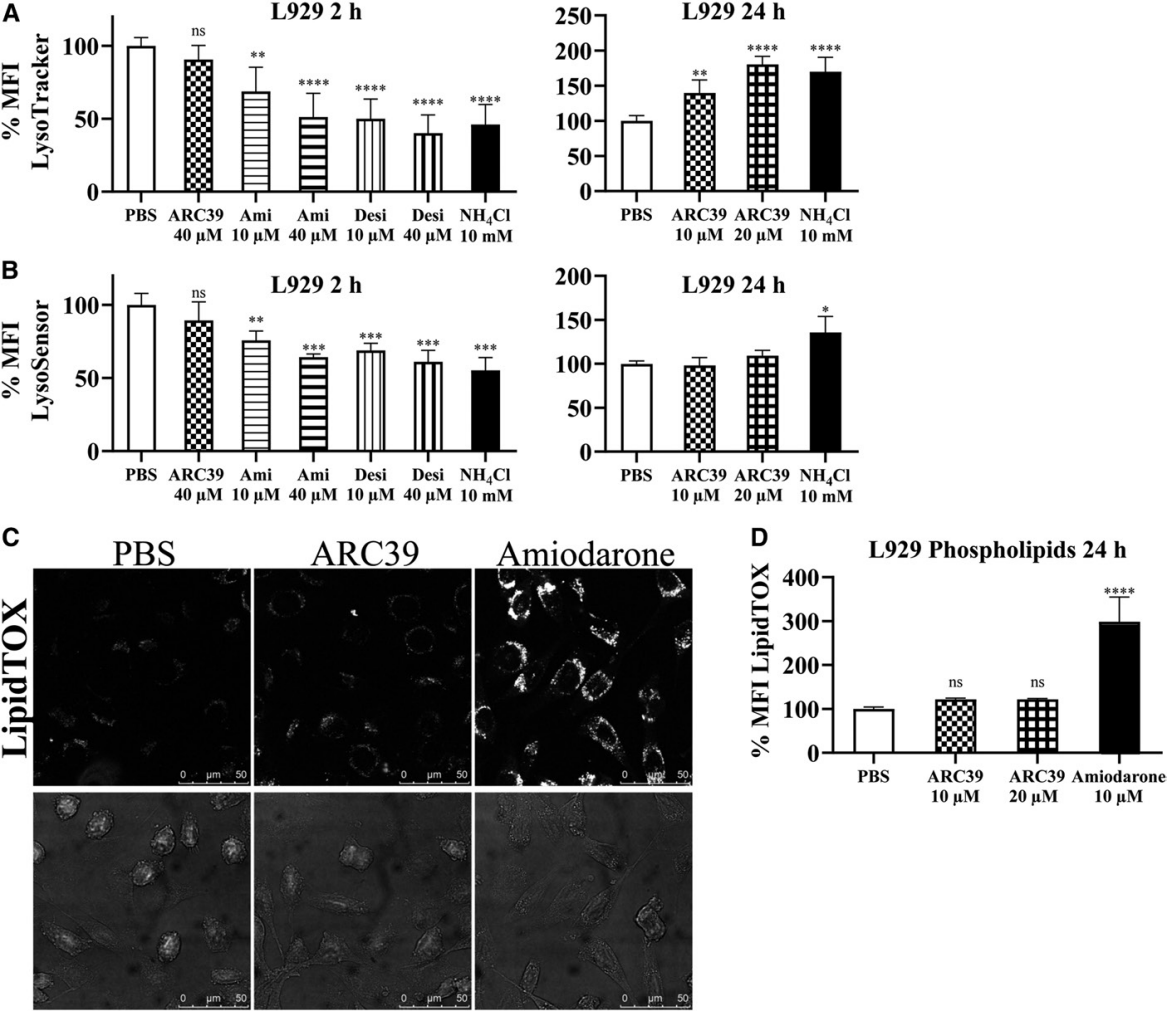
Effect of ARC39 on lysosomes. L929 cells were treated as indicated with 10 mM of NH_4_Cl as a positive control. A: LysoTracker Red (LTR) was added to a final concentration of 25 nM for 30 min at 37°C, and then the MFI of LTR was subsequently determined by flow cytometry. B: LysoSensor Blue DND-167 was added to a final concentration of 10 μM for 30 min at 37°C, and then its MFI was subsequently determined by flow cytometry. C: L929 cells were treated for 24 h with LipidTOX Green phospholipidosis detection reagent (1:1,000), together with PBS, 20 μM of ARC39, or 10 μM of amiodarone as a positive control, and then were analyzed with confocal microscopy for phospholipid accumulation (magnification ×100). D: After treatment for 24 h, as indicated, together with 1:1,000 LipidTOX Green, MFI was determined by flow cytometry for phospholipid accumulation. Control values are normalized to their mean. Data are represented as mean ± SD, n = 3 experiments. One-way ANOVA was used followed by Bonferroni correction. *P < 0.05, **P < 0.01, ***P < 0.001, ****P < 0.0001.

First, LysoTracker Red and LysoSensor Blue were used to assess the volume of the acidic compartment and pH, respectively. After 2 h, ARC39 had no significant effect on the MFI of either LysoTracker or LysoSensor, while amitriptyline and desipramine led to a reduction in the signal of both (**Fig. 7A**, B; supplemental Fig. S4). This is consistent with previous observations, because FIASMAs are weak bases and could lead to an initial alkalization of the lysosomes (19, 26). After 24 h, however, ARC39 increased the signal of LysoTracker only (Fig. 7A, B; supplemental Fig. S4), which could be attributed to the accumulation of sphingomyelin and a potential adaptive lysosomal response. Amitriptyline and desipramine led to an even more pronounced increase in the signal of LysoTracker after 24 h (not shown). The influence of many lysosomotropic drugs on the lysosomal compartment has been studied in detail elsewhere (43).

Next, we investigated to determine whether ARC39 could inhibit other lysosomal phospholipases and induce phospholipidosis. Using a LipidTOX reagent, which contains fluorescently labeled phospholipids, there was no significant accumulation of phospholipids 24 h after treatment compared with the positive control (Fig. 7C, D). Of note, there was an ∼20% increase in LipidTOX signal with ARC39 that did not show dose dependence between 10 and 20 μM.

### ARC39 in vivo

**Fig. 8.**
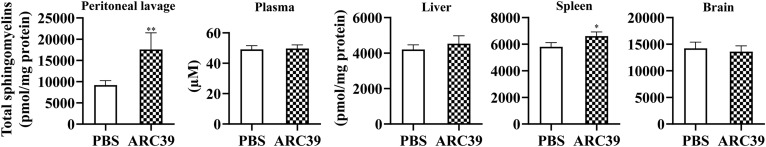
ASM inhibition by ARC39 in vivo. ARC39 was applied intraperitoneally every 12 h for 96 h at a dose of 0.16 mM 10 μl/g and sphingomyelin content was determined by MS 12 h after the last injection. Data are represented as mean ± SD, n = 4 mice per group. Unpaired Student’s *t*-test was used. *P < 0.05 **P < 0.01.

To test the potential effectiveness of ARC39 in the context of ASM inhibition in vivo, ARC39 was applied intraperitoneally in C57BL/6 WT mice. Importantly, at least for clodronate, it was reported that intraperitoneal absorption in mice was nearly complete judged by the drug concentration in bone, which was identical to intravenous administration (44). Knowing the pharmacokinetics of BPs, we assumed that high doses may be required to reach sufficient concentrations for ASM inhibition in organs. We, thus, undertook experiments to determine the maximal nontoxic dose both in a single-dose (supplemental Fig. S5) and a multiple-dose regimen (supplemental Fig. S6). Very high doses led to toxicity, particularly impaired renal function indicated by elevated blood urea nitrogen levels, liver damage indicated by elevated aspartate transaminase (glutamate-oxaloacetate transaminase) levels, and lethality. Acute renal toxicity in vivo of certain BPs was reported in rats (45) and in patients (46, 47). We then used subtoxic doses (corresponding to 0.32 mM 10 μl/g single dose and 0.16 mM 10 μl/g every 12 h for 96 h) to investigate ASM inhibition. Of note, these doses are still ∼5- to 10-fold higher than usually reported doses for other clinically relevant BPs. We first determined ASM activity after the single-dose or the short-term multiple-dose regimen with the conventional assay. No significant reduction was detected in either case except locally in the peritoneal lavage (not shown). Because the conventional assay may be subject to artifacts in this case because the organs are usually many-fold more diluted than cells during processing steps, we sought to quantify sphingomyelins by MS in the organs after the short-term treatment. A relevant and significant increase in total sphingomyelins was only observed locally in the peritoneal lavage (**Fig. 8**). Ceramides were not changed (not shown).

## DISCUSSION

Inhibition of ASM could be utilized as an investigational tool and potentially as a therapeutic intervention in the future (8, 9, 48). FIASMAs are widely used as inhibitors of ASM in vitro and in vivo and are clinically approved and generally well tolerated. However, FIASMAs act in an indirect manner and it has been shown, at least in vitro, that some of them may inhibit other enzymes involved in lipid metabolism and especially in sphingolipid metabolism. In vivo, FIASMAs lead to a relevant reduction of ceramide related to the inhibition of ASM and have been successfully used to reproduce the phenotype observed in ASM heterozygous mice in models of cystic fibrosis and major depression (4, 5).

It is desirable, at least for research purposes, to validate other more selective and potent ASM inhibitors. In this study, we characterized ARC39, which is one of the most potent ASM inhibitors developed so far (27). We show that ARC39 inhibits both L-ASM and S-ASM efficiently and selectively in vitro by directly blocking the catalytic activity without downregulating ASM mRNA or protein and, more importantly, works without inhibiting AC downstream of ASM and other acid hydrolases in the lysosomal sphingolipid catalytic pathways. Elojeimy et al. (19) reported that desipramine inhibits AC at 10 μM in vitro in a time-dependent manner. We also show that AC activity is partially inhibited with both amitriptyline and desipramine in L929 cells.

Because ARC39 is a BP, it is highly negatively charged at physiological pH. Thus, it is unlikely that passive diffusion through membranes occurs. The compound has been used in numerous studies to obtain outcomes related to ASM inhibition, e.g., it inhibited platelet-activating factor-induced pulmonary edema in isolated, ventilated, and perfused rat lungs (28), cellular stress response and death receptor signaling (34–37), and murine acute lung injury following transfusion of aged platelets in vivo when WT platelets had been stored with ARC39 (38). There was, however, no clear evidence whether this inhibitor could effectively reach the endo-lysosomal compartment and inhibit L-ASM because the majority of these biological effects are linked to S-ASM and/or surface activity of L-ASM upon translocation onto the outer leaflet of the plasma membrane. Here, we provide the following evidence of efficient L-ASM inhibition by ARC39 in vitro, as it led to: *1*) a dose- and time-dependent decrease in ASM activity determined with a FRET probe-based in situ assay in intact living cells; *2*) a dose-dependent increase of sphingomyelins and sphingomyelin/ceramide ratio after 24 h treatment as determined by MS; and *3*) lysosomal accumulation of exogenous BODIPY FL sphingomyelin.

The fact that ARC39 obviously acts in living cells is consistent with the early findings that BPs are able to enter the cytoplasm and other organelles (49). One particular study by Thompson et al. (50) confirmed the cellular uptake of alendronate and zoledronate (N-BPs) by fluid-phase endocytosis in the presence of Ca^2+^ and that these compounds accumulate in the endocytic vesicles. This mechanism of uptake could possibly explain the time-dependent ASM inhibition over h seen in L929 cells because BPs accumulate in the endo-lysosomal compartment with similar kinetics until reaching saturation.

ARC39 is relatively potent, and a final concentration of 0.3–0.5 μM in the assay is enough to inhibit ASM activity almost completely when adding the inhibitor directly to cell lysates (Fig. 1B). In contrast to that, a dose of 20 μM is required to inhibit ASM activity almost completely in living cells (Fig. 2A, B; supplemental Fig. S1). Of note, in contrast to the assays where lysates are treated with ARC39, in the assays where living cells are treated, two washing steps after incubating the compound with cells before lysis and a 1:10 dilution included in the micellar assay leads to a substantial dilution of ARC39 (see the Materials and Methods). The finding that inhibition with such a direct reversible inhibitor is still detectable even after these dilution steps in the conventional assay in cellular lysates supports the notion that the compound is potent and is taken up, potentially due to the proposed mechanism of uptake of BPs by endocytosis with accumulation in the endo-lysosomal compartment (50). However, we cannot fully exclude that ARC39 may have other effects on the enzyme.

We also provide an additional functional validation for ASM inhibition by ARC39, which abrogated platelet- and ASM-promoted adhesion of melanoma cells in vitro. This effect is, in turn, attributed to a platelet-derived zinc-dependent cell surface ASM activity and also supernatant activity (S-ASM) (9). Of note, when WT platelets had been stored with ARC39, transfusion of these platelets into recipient mice abrogated acute lung injury although the platelets were thoroughly washed before transfusion (38).

In vitro, the toxicity of ARC39 is generally low relative to the efficacy of ASM inhibition. BPs are widely used in the clinic as antiresorptive reagents to treat certain bone disorders like osteoporosis and to impede osteolytic lesions in cancer metastasis and multiple myeloma. Inhibition of FPP synthase is one major mechanism of action but also a cause of N-BP toxicity (39–42). Unlike ARC39, however, in which the -NH_2_ group is located in the R^1^ side chain, it is located in all other clinically relevant N-BPs in the R^2^ side chain. Dunford et al. (41) reported on the structure-activity relationship between the inhibition of FPP synthase in vitro and inhibition of bone resorption in vivo in N-BPs. In fact, Brown et al. (51) reported that replacing the hydroxyl group in the R^1^ chain by an amino group abolished or markedly reduced the antiresorptive potency of osteoclasts and the ability of both olpadronate and pamidronate but not etidronate to inhibit the growth of amoebae. Our data suggest that ARC39 could potentially inhibit FPP synthase and lead to unprenylated RAP1A accumulation. However, the potency and the relevance of this inhibition requires further investigation.

Using LysoTracker and LysoSensor staining, we showed that ARC39 affects the lysosomal compartment relatively minimally after 24 h, which may be attributed to ASM inhibition, sphingomyelin accumulation, and a potential lysosomal adaptive response. Of note, this increase of the MFI of LysoTracker after 24 h treatment with ARC39 or amitriptyline compared with vehicle treated was difficult to observe when cells had been cotreated for 24 h with exogenous BODIPY-sphingomyelin. A possible explanation is that feeding exogenous sphingomyelin to the cells could itself lead to an increase of the lysosomal volume regardless of the treatment.

More importantly, no significant accumulation of phospholipids was detected after 24 h with LipidTOX reagent upon treatment with ARC39.

However, the inhibitory effect of ARC39 on ASM in vitro could not be confirmed in vivo. When applying high subtoxic doses intraperitoneally, a relevant increase in sphingomyelins was only detected locally in the peritoneal lavage. As a BP, ARC39 may have high affinity to bone surfaces and could be very rapidly cleared from the plasma and soft tissues predominantly by bone and also by excretion in the kidney (52–55). On the other hand, there is currently no reliable method to determine ASM inhibition in vivo directly without possibly interfering with the enzyme-inhibitor interaction. Quantitating tissue sphingomyelin instead may provide useful information, although it may not always reflect ASM inhibition, particularly in a **s**hort-term treatment. In any case, local administration of such compounds, e.g., inhalation, may still be an option.

In conclusion, these data suggest that ARC39 is a potent and specific ASM inhibitor in vitro. There is, however, still a need for improved compounds suitable for systemic administration in vivo that can reach tissue concentrations relevant to ASM inhibition with lower doses. One promising approach to circumvent the need for high doses and the problem of biséphosphonate pharmacokinetics could be a new ARC39 prodrug that can be cleaved intracellularly into the active ARC39. This compound is currently being developed. Nanoparticle coating for targeted tissue delivery in vivo could be another possible strategy (56). This last option is particularly worth considering when targeting the compound to the central nervous system because BPs do not bypass the blood brain barrier, which could be utilized as a novel therapeutic option in a model of Farber disease, a rare lysosomal storage disorder, where a recent proof-of-concept study suggested that decreasing Asm activity is a potential therapeutic option (57).

## Supplementary Material

Supplemental Data
