# Supplementary material for: Characterization of the small molecule ARC39, a direct and specific inhibitor of acid sphingomyelinase in vitro
Source: J Lipid Res. 2020 Mar 10;61(6):896–910. doi: 10.1194/jlr.RA120000682 (PMC7269768; doi:10.1194/jlr.RA120000682)
Supplement: Supplemental Data [file supp_61_6_896__index.html]

Characterization of the small molecule ARC39, a direct and specific inhibitor of acid sphingomyelinase in vitro — ARC39 a direct inhibitor of acid sphingomyelinase — Characterization of the small molecule ARC39, a direct and specific inhibitor of acid sphingomyelinase in vitro — Supplemental Data 

# Characterization of the small molecule ARC39, a direct and specific inhibitor of acid sphingomyelinase in vitro

## Supplemental Data

- Supplemental Data - Supplemental Data
