# Supplementary material for: Characterization of the small molecule ARC39, a direct and specific inhibitor of acid sphingomyelinase in vitro
Source: J Lipid Res. 2020 Mar 10;61(6):896–910. doi: 10.1194/jlr.RA120000682 (PMC7269768; doi:10.1194/jlr.RA120000682)
Supplement: Supplemental Data [file supp_RA120000682_158760_1_supp_487247_q6q3t6.pdf]

Supplemental figures:

Characterization of the small molecule ARC39, a direct and specific inhibitor of acid sphingomyelinase *in vitro*

Eyad Naser[1], Stephanie Kadow[1], Fabian Schumacher[1,2], Zainelabdeen H. Mohamed[3,4], Christian Kappe[3], Gabriele Hessler[1], Barbara Pollmeier[1], Burkhard Kleuser[2], Christoph Arenz[3], Katrin Anne Becker[1], Erich Gulbins[1,5], Alexander Carpinteiro[1,6]

[1]Department of Molecular Biology, University of Duisburg-Essen, Hufelandstraße 55, 45147 Essen, Germany.

[2]Department of Toxicology, Institute of Nutritional Science, University of Potsdam, Arthur-Scheunert-Allee 114-116, 14558 Nuthetal, Germany.

[3]Institute of Chemistry, Humboldt University of Berlin, Brook-Taylor-Straße 2, 12489 Berlin, Germany.

[4]Medicinal Chemistry Department, Faculty of Pharmacy, Assiut University, Assiut 71526, Egypt.

[5]Department of Surgery, University of Cincinnati, 231 Albert Sabin Way, ML 0558, Cincinnati, Ohio 45229, USA.

[6]Department of Hematology, University Hospital Essen, Hufelandstraße 55, 45147 Essen, Germany.

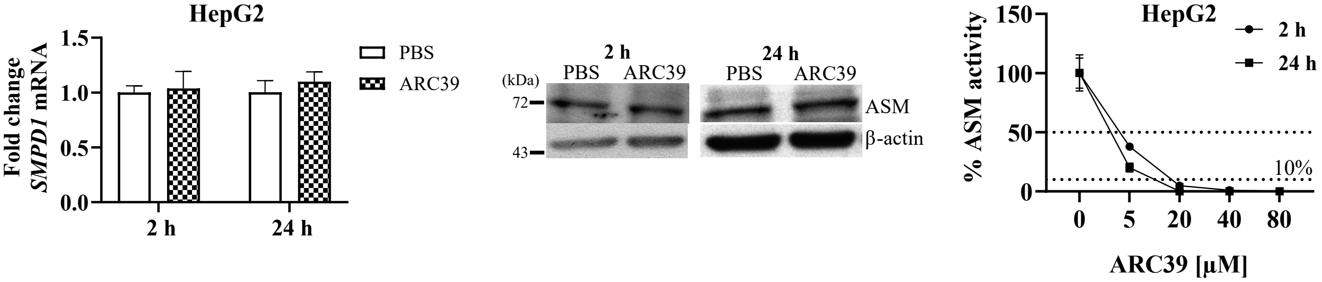

Supplemental Figure S1. *Left:* Fold change of *SMPD1* mRNA relative to *HPRT1*, ASM protein level (representative blot) after treatment with 20 µM ARC39 as indicated in HepG2 cells. *Right:* ASM enzyme activity. Enzyme activity is normalized to the protein concentration and control values are normalized to their mean. L-ASM activity here was determined with the conventional ASM assay (without $ZnCl_2$ in the assay buffer). Data represented as mean ± SD, n=3 experiments. Two-way ANOVA was used. $P<0.05$ for time as a source of variation in ASM activity.

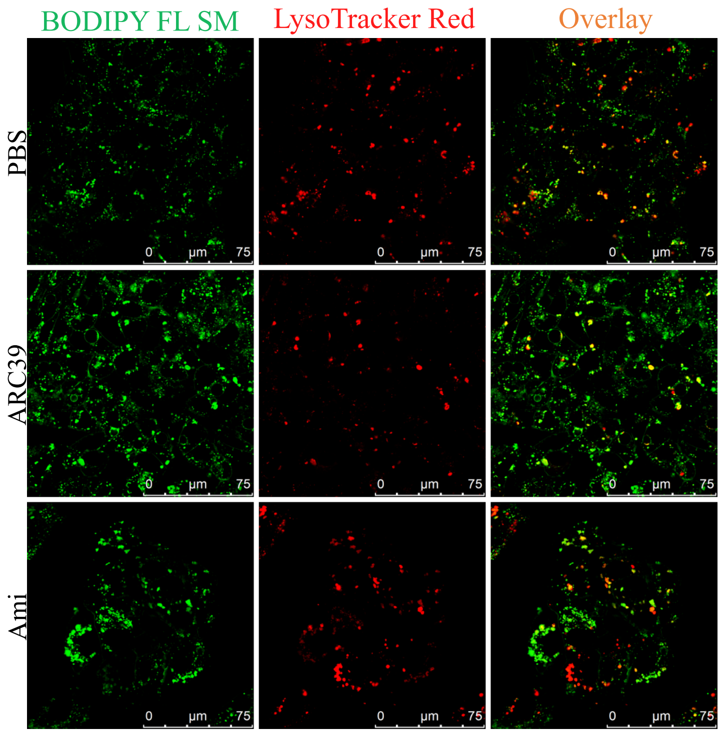

Supplemental Figure S2. ARC39 leads to lysosomal accumulation of exogenous sphingomyelin. HepG2 cells were incubated for 24h with 1 µM BODIPY FL sphingomyelin (SM) together with PBS as control, 10 µM ARC39 or 10 µM amitriptyline (Ami) as a positive control. Then, they were incubated 30 min at 37°C with 25 nM LysoTracker Red DND-99. Fresh medium was added then the cells were analyzed by confocal microscopy, magnification 100×.

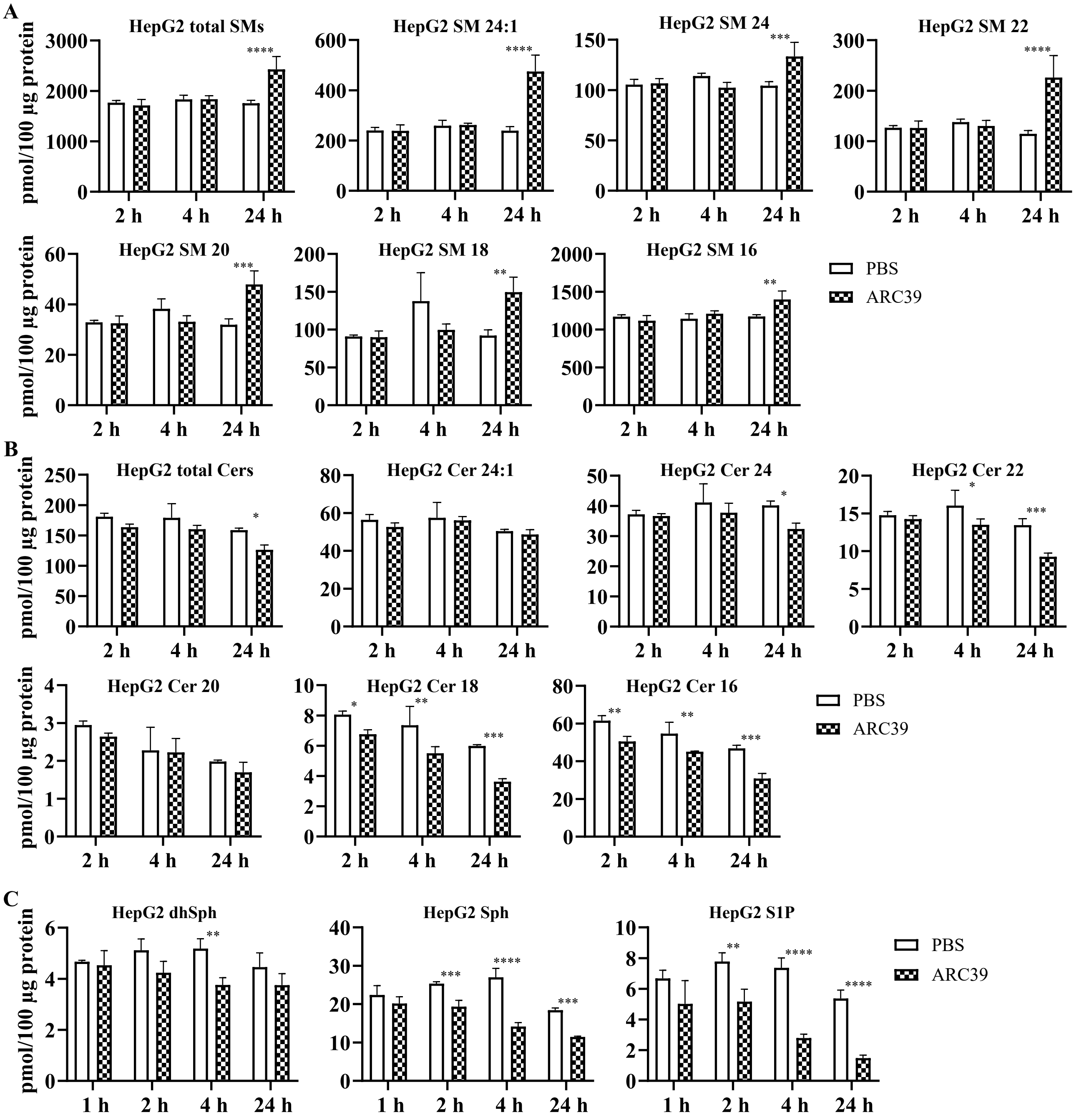

Supplemental Figure S3. Sphingolipidomic analysis after treatment with ARC39 in HepG2 cells. Cells were treated with 20 µM ARC39 and at the indicated time points the endogenous levels of the following sphingolipids were determined by mass spectrometry in whole-cell lysates. **(A)** Sphingomyelins (SMs). **(B)** Ceramides (Cers). Numbers indicate the chain length and saturation of the fatty acyl chain. **(C)** Dihydrosphingosine (dhSph), Sphingosine (Sph) and sphingosine-1-phosphate (S1P), respectively. Data represented as mean ± SD, n=3 experiments. Two-way ANOVA was used followed by Bonferroni correction. **P*<0.05 ***P*<0.01 ****P*<0.001 *****P*<0.0001.

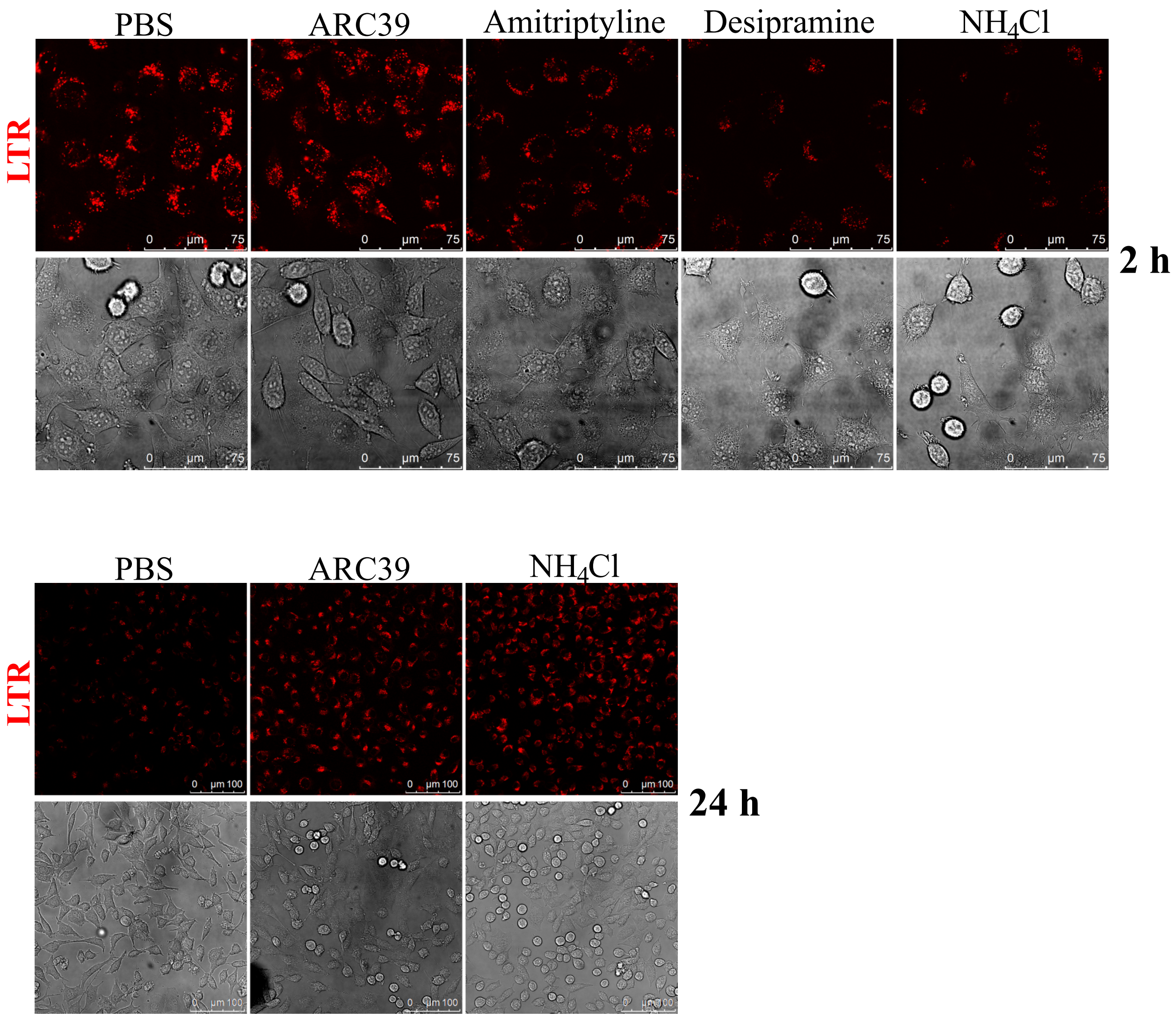

Supplemental Figure S4. Effect of ARC39 on lysosomes. L929 cells were treated 2 h (up) or 24 h (down) with PBS, 40 µM ARC39 for 2 h, 20 µM ARC39 for 24 h, 10 µM amitriptyline (2h), 10 µM desipramine (2h) or 10 mM NH_4Cl as a positive control. Then, LysoTracker Red (LTR) was added to a final concentration of 25 nM for 30 min at 37°C. Fresh medium was added and the cells were analyzed by confocal microscopy. Magnification 100× (up) and 40× (down).

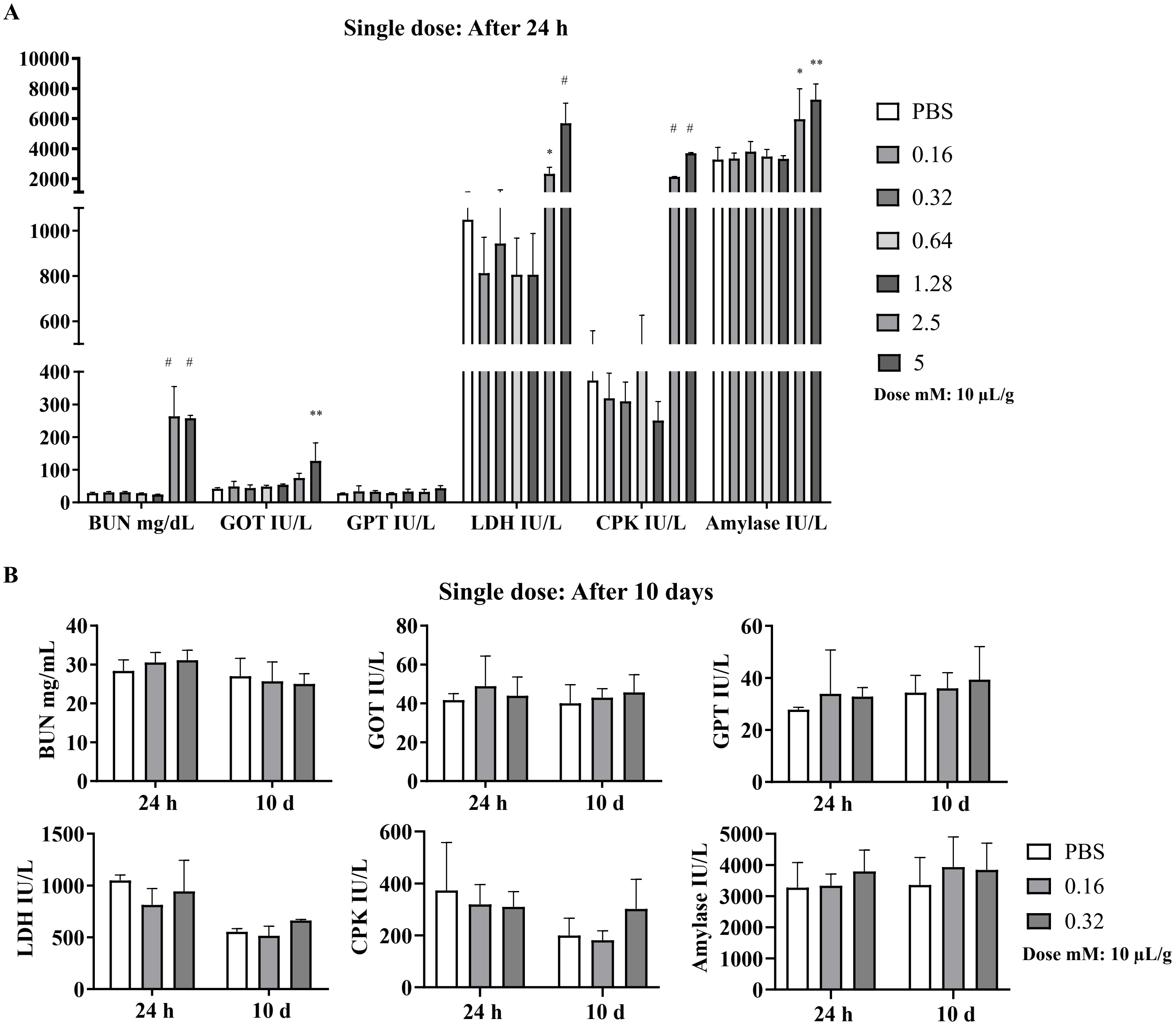

Supplemental Figure S5. Toxicity of ARC39 *in vivo* in a single-dose regimen. The compound was applied i.p. for one time at the indicated doses. After 24 h in **(A)** and after 10 d in the same individuals in **(B)**, serum was collected and the following serological markers were assessed: Blood urea nitrogen **(BUN)**, glutamate-oxaloacetate transaminase **(GOT)** / aspartate transaminase, glutamate-pyruvate transaminase **(GPT)** / alanine transaminase, lactate dehydrogenase **(LDH)**, creatine phosphokinase **(CPK)** and amylase. On day 10, only the three groups that did not exhibit morbidity or lethality between 24 h-10 d are shown. Data represented as mean ± SD, n=3 mice per group. One-way ANOVA was used in (A) and two-way ANOVA was in (B), both followed by Bonferroni correction. *P<0.05 **P<0.01 #P<0.0001.

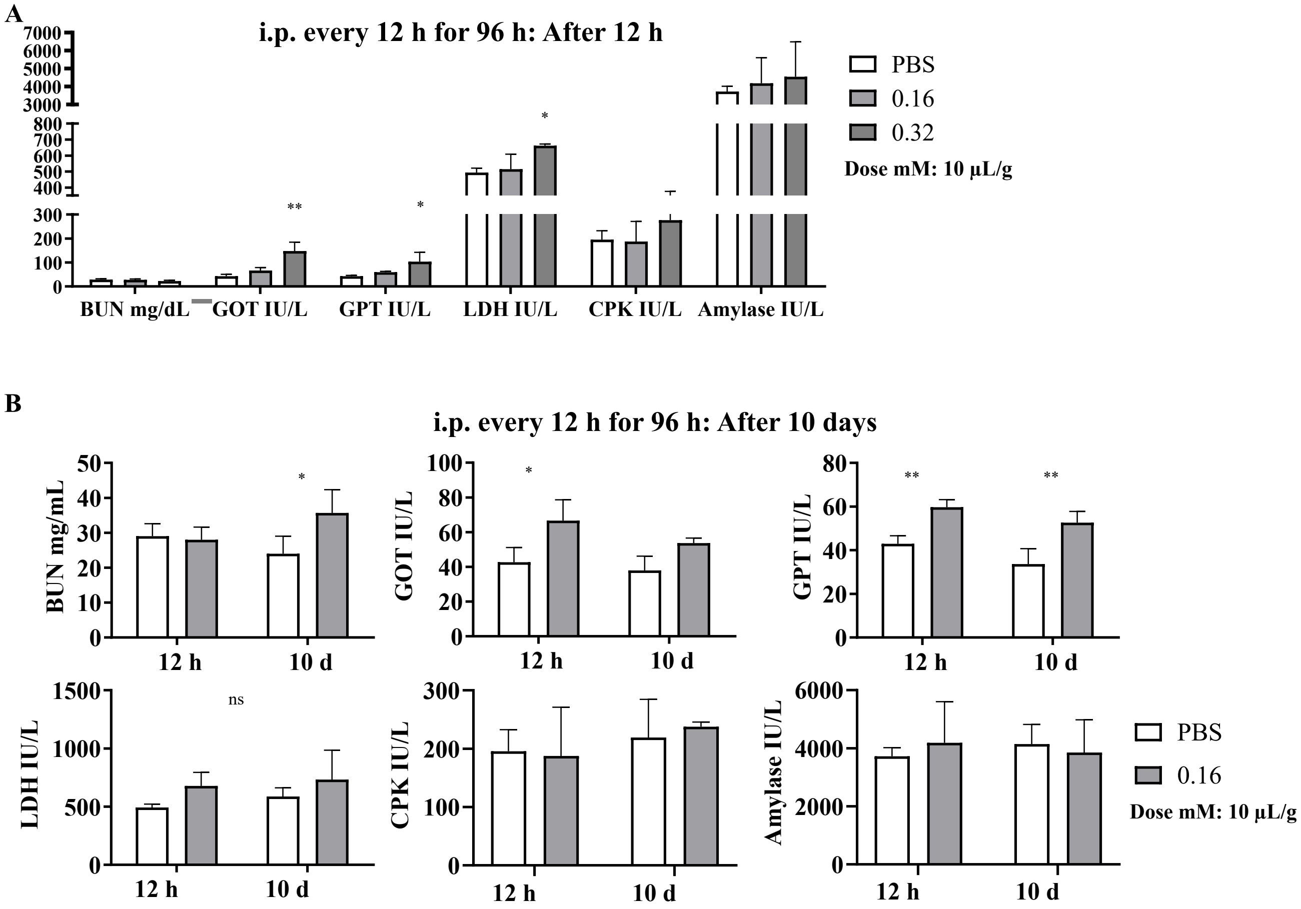

Supplemental Figure S6. Toxicity of ARC39 *in vivo* in a multiple-dose regimen. The compound was applied i.p. every 12 h for 96 h. After 12 h in **(A)** and after 10 d in the same individuals in **(B)**, serum was collected and the following serological markers were assessed: Blood urea nitrogen **(BUN)**, glutamate-oxaloacetate transaminase **(GOT)** / aspartate transaminase, glutamate-pyruvate transaminase **(GPT)** / alanine transaminase, lactate dehydrogenase **(LDH)**, creatine phosphokinase **(CPK)** and amylase. On day 10, only the two groups that did not exhibit morbidity or lethality between 12 h-10 d are shown. Data represented as mean ± SD, n=3 mice per group. One-way ANOVA was used in (A) and two-way ANOVA was in (B), both followed by Bonferroni correction. *P<0.05 **P<0.01.
